# Assessing Temporal Stability for Coarse Scale Satellite Moisture Validation in the Maqu Area, Tibet

**DOI:** 10.3390/s130810725

**Published:** 2013-08-16

**Authors:** Haris Akram Bhatti, Tom Rientjes, Wouter Verhoef, Muhammad Yaseen

**Affiliations:** 1 Department of Water Resources, Faculty of Geo-Information Science and Earth Observation (ITC), University of Twente, Hengelosestraat 99, 7514 AE Enschede, The Netherlands; E-Mails: t.h.m.rientjes@utwente.nl (T.R.); verhoef@itc.nl (W.V.); m.yaseen@utwente.nl (M.Y.); 2 Department of Civil Engineering, NED University of Engineering and Technology, Karachi 75270, Pakistan; 3 Centre of Excellence in Water Resources Engineering, University of Engineering and Technology, Lahore 54890, Pakistan

**Keywords:** temporal stability, representative mean soil moisture (RMSM), correlation, advance microwave scanning radiometer (AMSR-E), satellite validation

## Abstract

This study evaluates if the temporal stability concept is applicable to a time series of satellite soil moisture images so to extend the common procedure of satellite image validation. The area of study is the Maqu area, which is located in the northeastern part of the Tibetan plateau. The network serves validation purposes of coarse scale (25–50 km) satellite soil moisture products and comprises 20 stations with probes installed at depths of 5, 10, 20, 40, 80 cm. The study period is 2009. The temporal stability concept is applied to all five depths of the soil moisture measuring network and to a time series of satellite-based moisture products from the Advance Microwave Scanning Radiometer (AMSR-E). The *in-situ* network is also assessed by Pearsons's correlation analysis. Assessments by the temporal stability concept proved to be useful and results suggest that probe measurements at 10 cm depth best match to the satellite observations. The Mean Relative Difference plot for satellite pixels shows that a RMSM pixel can be identified but in our case this pixel does not overlay any *in-situ* station. Also, the RMSM pixel does not overlay any of the Representative Mean Soil Moisture (RMSM) stations of the five probe depths. Pearson's correlation analysis on *in-situ* measurements suggests that moisture patterns over time are more persistent than over space. Since this study presents first results on the application of the temporal stability concept to a series of satellite images, we recommend further tests to become more conclusive on effectiveness to broaden the procedure of satellite validation.

## 1. Introduction

Soil moisture is a fundamentally important variable in the hydrological and energy cycle. In the hydrological cycle, moisture influences processes such as infiltration, recharge, but also generation of runoff processes such as interflow and overland flow [1]. In the energy cycle, soil moisture separates net radiation in sensible and latent heat that causes evapotranspiration at the land surface. Characteristic to soil moisture is that moisture contents vary over the space and time domains by aspects and factors that relate to topographic variability, soil texture and land cover [2,3]. In general, *in-situ* moisture observations best relate to each other when stations are located at close distance and when observation intervals are small. Moisture may be observed by *in-situ* methods or by satellite missions such as the Advanced Microwave Scanning Radiometer—Earth Observing System (AMSR-E) (http://sharaku.eorc.jaxa.jp/AMSR/index.html), the Soil Moisture and Ocean Salinity (SMOS) satellite mission (www.esa.int/esaLP/LPsmos.html) and the METOP ASCAT (The Meteorological Operational satellite programme Advanced SCATterometer) satellite mission (http://www.esa.int/esaME/ascat.html). *In-situ* methods commonly rely on field measurements by means of a network of moisture probes. Probes measure systematically over time following the design of the network. In general, *in-situ* measurements are at the point scale (<1 m^2^), satellite observations are at the pixel footprint scale that, for instance, may be as large as 25 km × 25 km for AMSR-E or 50 km × 50 km for the SMOS satellite mission. Moreover, satellite observations provide information for shallow (<5 cm) land surface depths due to limited penetration [4]. In the time domain, field measurements commonly are at high temporal resolution (e.g., 15 min. interval) whereas satellites observe moisture mostly once per day. These characteristics, among aspects that relate to differences in probe and satellite sensor technologies, suggest that any comparison is not straightforward. For this reason, satellite observations require validation to indicate how well satellite estimates match with the field (*i.e.*, *in-situ*) measurements.

Validation is imperative in order to make any conclusions about the reliability of satellite observations [5] before being used in the practice of water management. Moreover, soil moisture at a local scale can be measured with a certain degree of certainty but it is rather complex to upscale measurements to larger spatial scales [6]. Principal assumptions to the validation of a satellite image are that field measurements and satellite observations may be compared and that, in addition, validation results apply to all satellite pixels, although for most pixels field measurements are not available. So in essence, there is no guarantee that satellites observe moisture patterns correctly. This makes validation of satellite observed moisture an important and challenging task, particularly when moisture is characterized by high variability.

In satellite validation, it is common to compare satellite observations to field (*i.e.*, *in-situ*) measured counterparts where assessments rely on scatter plots, time series comparison and on statistical analysis [7–12]. Ideally, the comparison should be done at homogeneous and uniform spatial domains so that probe measurements can be related uniquely to satellite observations. Clearly when pixel sizes increase, the assumption of uniformity at pixel scale weakens. Pixels comprise a mixture of different land covers, soil textures and topographic characteristics and thus any moisture estimate at the pixel foot print scale represents a spatial average. It is common to weigh *in-situ* measurements for the relative size of the various strata to arrive at a weighted spatial average moisture estimate. We refer to Dente *et al.* [13] for such application in the Maqu validation site that we also selected for this study.

Validation of satellite observations commonly relies on: (i) scatter plots where field measured moisture is plotted against satellite-based soil moisture to indicate how well values match [13] and (ii) on time series inter-comparison for the observation period [8,9]. Whereas scatter plots assess the overall relation, time series comparison allows for identification of periods with large and/or small deviations. Quantitative analyses often rely on statistical indices such as the Root Mean Square Error (RMSE), Bias and correlation coefficient [10–12]. Low values for RMSE and Bias and high correlation values suggest that satellite estimates match well to *in-situ* measured counter parts. These assessments seem straightforward but there are a number of weaknesses. For instance, conclusions on validation may be doubtful when: (i) only a small number of stations is available (ii) time series are too short to represent the natural variability; (iii) instrumentation is inaccurate or (iv) non-representative measurement depths (to deep or too shallow) have been selected. For brevity, we ignore a full description on each of these aspects and refer readers to [9,14–16] on these validation issues.

Challenging to validation is: (i) to understand the content of the information that is embedded in the *in-situ* measured time series that serve to represent the time-space moisture patterns and (ii) how that information can be utilized to better understand the importance of each measurement site to validate the satellite observations. In this respect, we refer to Vachaud *et al.* [17] who proposed the concept of temporal stability that aims to assess information collected by moisture observation networks. In the same work, the concept is referred to as ‘the time invariant association between spatial location and classical statistical parametric values’ and based on the idea that a soil moisture field maintains its spatial pattern over time with one station that represents the area mean moisture. In the approach, such station is referred to as the “Representative Mean Soil Moisture (RMSM) station”. The mean values for the remaining stations serve to characterize stations from driest to wettest and indicate the Mean Relative Difference (MRD) for each station. In the analysis for each station, the standard deviation (SD) of the time series is estimated that indicates variability in the time domain. The temporal stability concept has applications to areas of varying sizes that range from a few meters [18] to a few hectometers [19], and areas of size less than 1 ha [17,20] to a few hectares [21,22]. An application to an area larger than 3,000 km^2^ is shown in Dente *et al.* [13]. Applications to a time series of satellite images are not known to the authors.

A second method to assess characteristics of temporal stability is correlation analysis [23,24] as described in Cosh *et al.* [25]. Correlation analysis serves to measure dependency between two sample variables with Spearman rank correlation and/or Pearson product-moment correlation applied in soil moisture studies. Spearman's correlation has been applied in [26–29] to assess temporal characteristics of soil moisture to indicate persistence of ranks over consecutive days. Pearson's correlation method measures the stability of moisture patterns over consecutive time instants across the network as well as between stations for the same instants in time. Cosh *et al.* [24,25,30] applied the temporal stability concept and pearson correlation analysis to evaluate soil moisture measuring networks. These studies concluded that averaged moisture estimates of an area can be represented by a single station. Our work extends on the satellite validation study by Dente *et al.* [13] who reported on temporal stability analysis for measurements at shallow depth (5 cm) for the Maqu area. In their study, moisture variability on a seasonal basis was assessed and it was concluded that temporal stability in the dry season (winter period) is higher than in monsoon and transition periods. Further, the spatial patterns of soil moisture are not always stable, as some stations are wetter than average in one season and dryer in other seasons. In our approach, we assess temporal stability of the *in-situ* network stations for all five probe depths (5, 10, 20, 40 and 80 cm) and also apply Pearson's correlation analysis. Our work extends on earlier studies by Cosh *et al.* [25,30] as we evaluate applicability of the temporal stability analysis to a time series of satellite images. We use all grid pixels to represent a spatial sample that we consider to represent a network of probes. Analyzing the pixel estimates for the time series should result in a single pixel that indicates the RMSM for the time series of images. The premise of this approach is that estimates by probes are comparable to estimates by satellites, that is principle to satellite validation. We defined the following objectives for this study: (a) to identify the RMSM station for respective probe depths and to assess the probe depth that is best suited for satellite validation (b) to evaluate how well the AMSR-E soil moisture observations match observations of the RMSM stations and to evaluate if a RMSM pixel can be identified.

This paper is organized as follows: in Section 2, the study area and data are presented. The applied methodology is described in Section 3, which is divided in three sub-sections: the temporal stability concept, correlation analysis and time series analysis. Hereafter, in Section 4 the results are described and discussed and in Section 5 conclusions are drawn.

## 2. Study Area and Data

For this study, the Maqu area is selected. It is situated in the northeastern part of the Tibetan Plateau and located southeast of Maqu city, on the border between Gansu and Sichuan provinces, China (33°30′–34°15′ N latitude, 101°38′–102°45′ E longitude). The area has an elevation ranging from 3,160–4,664 m.a.s.l. and is characterized by the river valleys of the Yellow River, the Black River and the Lang River mountain ranges towards the area divide. Topographically, the Maqu area is characterized by hills, valleys, rivers, wetlands, grassland and bare areas with uniform land cover of short grassland and some wetlands. Soil texture mostly is silt loam with organic matter that is higher in wetland areas than in grassland areas. According to the Köppen Classification System [31], the region has a continental climate, with dry and cold winters and cool and rainy summers.

### 2.1. In-Situ Data

In the Maqu area, a soil moisture and soil temperature monitoring network of 20 stations has been installed by Cold and Arid Regions Environmental and Engineering Research Institute, Chinese Academy of Sciences (CAREERI, CAS) and ITC (Faculty of Geo-Information Science and Earth Observation, University of Twente, Enschede, The Netherlands). Stations record and store data at 15 min interval, which serve for validation of SMOS, ASCAT and AMSR-E satellite missions.

**Figure 1.**
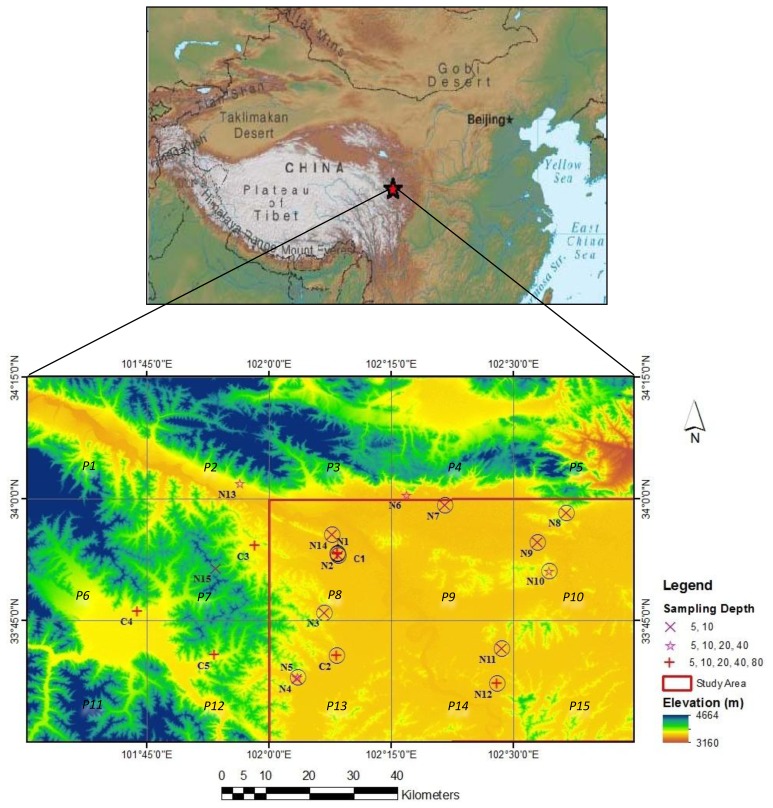
A Shuttle Radar Topography Mission (SRTM) digital elevation model (DEM) of Maqu Area. Station locations are indicated by various symbols. P1, P2,…..P15 represents pixel numbers. Stations selected for analysis are indicated by circles. The red box marks the pixels (*P8, P9, P10, P13, P14, P15*) that are selected for satellite validation.

The network covers an area of approximately 80 km × 40 km. Station locations have been selected to monitor the area at different altitudes and slopes with variable land cover and soil type [13]. Figure 1 shows a Shuttle Radar Topography Mission (SRTM) digital elevation model (DEM) of the area with station locations indicated by different symbols according to the number of probes. Eleven stations are installed in the valleys of the Yellow River and Black River (C1, C2 N1, N2, N6, N7, N8, N9, N12, N13, and N14), three stations in the valleys between hills (C3, C4, C5), four stations on steep hill slopes (N3, N5, N10, N15) and two stations in wetlands (N4, N11).

During the installation of the stations, soil samples were collected to analyze bulk density, particle size distribution and organic matter content. Most of the stations are installed in silt loam soils, except for stations N9 and N10 that are characterized by sandy loam and loam-silt soils, respectively. The wetland stations N4 and N11 have the highest organic matter content (>130 g/kg), all the other stations are installed in areas with low organic content (<60 g/kg).

For each station, a number of soil moisture and soil temperature probes are installed. Stations C1-5, N1, N12 have measuring probes at 5, 10, 20, 40 and 80 cm depth, stations N5, N6, N10, N13 have probes till 40 cm depth whereas stations N2, N3, N4, N7, N8, N9, N11, N14, N15 collect data only at 5 and 10 cm depth.

Each station is equipped with an Em50 ECH20 data logger that records the soil moisture data at 15 min interval of the ECH2O EC-TM probes. As part of the installation of the network, recorded soil moisture data has been compared and calibrated against volumetric soil moisture measurements obtained by gravimetric sampling at all station locations. After calibration, the root mean square difference was found to be 0.02 m^3^·m^−3^[12,13,32] which suggests a good measurement accuracy of the moisture probes. For this study, time series for the year 2009 are used after screening and correction.

All soil moisture time series are screened for moisture values higher than the porosity value (0.55) of the shallow silt loam soil layer in the Maqu area. Time series from five stations (C2 at 80 cm, N4 at 5 and 10 cm, N5 at 5 cm and N15 at 5 cm) were excluded from further use since measurements systematically indicated moisture contents much higher than the maximum possible (*i.e.*, porosity value). For a more comprehensive description on the Maqu study site and the purpose of the installed network, we refer to [12,13,32].

### 2.2. Satellite Data

For this study, we use daily time series of the soil moisture products from the AQUA AMSR-E sensor as post-processed by the Vrije Universiteit Amsterdam (VUA) in The Netherlands and the National Aeronautics and Space Administration (NASA), in the USA [33,34]. The AMSR-E product has been applied in various studies where consistency and a good match were shown to observation networks [8–10]. In the following, we refer to the product by AMSR-E VUA for which images were downloaded from http://www.geo.vu.nl/∼jeur/lprm. Time series of AMSR-E VUA are selected for the year 2009 to match the period for which the network measurements are available (January–December, 2009). Following Owe *et al.* [35] for our analysis, we considered images from the descending overpasses that take place during night time (3:00–4:00 a.m.). Also, comparison for ascending and descending satellite observations with field measured soil moisture have shown better match with the latter one [8]. This is due to the assumption used in the retrieval algorithm that the surface temperature is closest to the soil temperature, which is more likely to happen during night time [13]. Image pixels that overlay the study area are numbered from left to right (column 1 to 5) and from top to bottom (row 1 to 3) with pixel numbers P1-5, P6-10 and P11-15 for respective rows.

Time series of all pixels are screened for values higher than the porosity value of the shallow soil layer. Results of screening indicated that time series of pixels P1, P2, P5, P6, P7, P11 are unreliable. We noticed that all these pixels overlay hilly terrain and signals that time series of the remaining (few) pixels that overlay hilly terrain may be doubtful as well. As such for application of the temporal stability concept, we also excluded time series of pixels P3, P4 and P12 and only used time series of pixels P8, P9, P10, P13, P14 and P15 that overlay flat terrain. Table 1 shows which stations are overlain by certain pixel.

## 3. Methodology

### 3.1. Temporal Stability Concept

For assessing and comparing statistics of the time series of the network stations, a MRD plot [17] is constructed. In such a plot, stations are ranked based on their MRD values that are estimated with reference to the network average soil moisture. Ranking of stations is from low (negative) to high (positive) MRD value where the station with the value closest to zero is the RMSM station. Low MRD values indicate that the averaged moisture is lower than the area Mean Soil Moisture (MSM) and vice versa. Introduced by Vachaud *et al.* [17], the MRD reads:

(1) $${\bar{\delta }}_{i}=\frac{1}{m}\sum_{j=1}^{m}\delta_{ij}$$

where *δ̅_i_* is the MRD at location *i*, *m* is the number of sampling days and *δ_ij_* is the relative difference at location *i* on day *j* and reads:

(2) $$\delta_{ij}=\frac{S_{ij}-{\bar{S}}_{j}}{{\bar{S}}_{j}}$$

where *S_ij_* is the soil moisture at location *i* on day *j* and *S̅_j_* is the average soil moisture on day *j* and reads:

(3) $${\bar{S}}_{j}=\frac{1}{N}\sum_{j=1}^{m}S_{ij}$$

where *N* is the number of sample locations. The temporal stability of each station is further characterized by the standard deviation (SD) of the MRD, SD(MRD), which indicates temporal variability of the moisture observations at each station. Stations that have low SD(MRD) value indicate low variability in the time series whereas high SD(MRD) indicates high temporal variability. Stations with low SD(MRD) commonly are termed temporally stable stations [25]. The SD(MRD) at location *i* reads:

(4) $$\text{SD}(\text{MRD})=\sqrt{\frac{{\left(\delta_{ij}-{\bar{\delta }}_{i}\right)}^{2}}{m-1}}$$

MRD plots are prepared for each of the five probe depths and for time series of AMSR-E VUA satellite images. For the latter, daily images are ordered chronologically and the MRD value is calculated for each pixel. The satellite-based MRD plot identifies the pixel that indicates the area RMSM. The station based MRD plots at four probe depths (5, 10, 20 and 40 cm) are evaluated for persistence to serve for satellite validation.

### 3.2. Correlation Analysis

Following Cosh *et al.* [24], we applied Pearson's correlation coefficient (*r_j,j_*_′_) to assess correlation of moisture patterns over time:

(5) $$r_{j,j^{\prime }}=\frac{\sum_{i=1}^{n}\left(S_{i,j}-{\bar{S}}_{\cdot ,j})(S_{i,j^{\prime }}-{\bar{S}}_{\cdot ,j^{\prime }}\right)}{\sqrt{\sum_{i=1}^{n}{\left(S_{i,j}-{\bar{S}}_{\cdot ,j}\right)}^{2}}\sqrt{\sum_{i=1}^{n}{\left(S_{i,j^{\prime }}-{\bar{S.}}_{,j^{\prime }}\right)}^{2}}}$$

where *S_i_*,*_j_* and *S_i_*,*_j_*_′_ are soil moisture observations for station i for given time instants *j* and *j*′ (e.g., days) respectively and *n* is number of stations. The average soil moisture content for time instant *j* for all stations is *S̅*_·,_*_j_*.

A second application of Pearson's correlation coefficient (*r_i_*,*_i_*_′_) is shown in Cosh *et al.* [25,30] where soil moisture stability is assessed between stations and reads:

(6) $$r_{i,i^{\prime }}=\frac{\sum_{j=1}^{m}\left(S_{i,j}-{\bar{S}}_{\cdot ,j}\right)\left(S_{i^{\prime },j}-{\bar{S.}}_{,j}\right)}{\sqrt{\sum_{j=1}^{m}{\left(S_{i,j}-{\bar{S}}_{\cdot ,j}\right)}^{2}}\sqrt{\sum_{j=1}^{m}{\left(S_{i^{\prime },j}-{\bar{S.}}_{,j}\right)}^{2}}}$$

where *S_i_*,*_j_* and *S_i_*_′_,*_j_* are soil moisture observations from two stations *i* and *i*′ for a given time instant *j*. The resulting coefficients indicate spatial dependency of stations. Values of *r_i,i_*_′_ range between +1 and −1 with uncorrelated stations having *r_i_*,*_i_*_′_ value close to 0. In Cosh *et al.* [30], high correlation is suggested when |*r_i_*,*_i_*_′_| > 0.7 which indicates stable moisture patterns by the network. Low correlation is suggested when |*r_i_*,*_i_*_′_| > 0.3 which indicates that moisture patterns differ in the network. In this study, we adopted these value ranges.

### 3.3. Time Series Analysis

We inter-compared station and satellite data for identification of periods with large and/or small deviations. We calculated the RMSE and Bias (Equations (7) and (8), respectively) for time series of network averages (*i.e.*, the field measurements) and time series of the RMSM stations, the RMSM pixel, image averages and pixels that overlay a station:

(7) $$\text{RMSE}=\sqrt{\sum_{j=1}^{m}\frac{{\left(Sg_{j}-Sc\right)}^{2}}{m}}$$

(8) $$\text{Bias}=\frac{1}{m}\sum_{j=1}^{m}\left(Sg_{j}-Sc\right)$$

where *Sg* is the network averaged soil moisture and *Sc* is the average of the variable selected for comparison. Note that *j* indicates the time instant with *m* the number of days. RMSE and Bias values near zero suggest that time series match well. Negative Bias indicates that the network shows higher moisture content than the variable selected for comparison and vice versa.

## 4. Results and Discussion

### 4.1. Temporal Stability Concept

**Figure 2.**
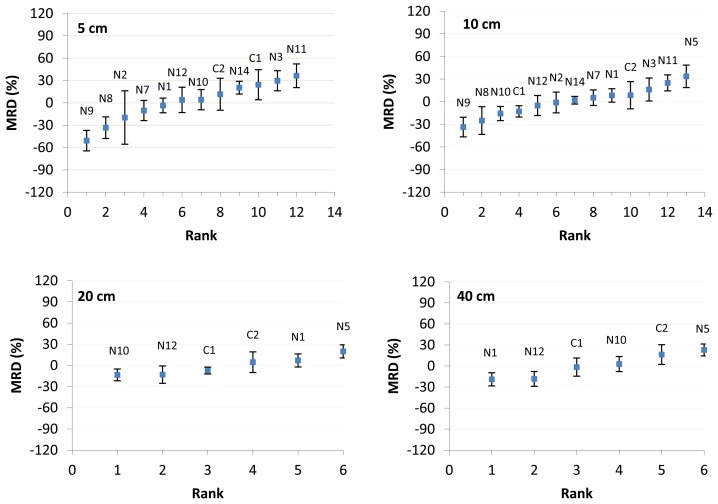
MRD plots at 5, 10, 20 and 40 cm depth. For each station the MRD is indicated by the small box. Whiskers indicate the standard deviation (SD) of the time series (SD(MRD)) for the year 2009. Station numbers refer to stations as shown in Figure 1.

MRD plots for respective moisture probe depths (Figure 2) shows that for each depth a specific RMSM station can be identified that indicates MSM: N1 for 5 cm, N2 for 10 cm, C2 for 20 cm and C1 for 40 and 80 cm.

For RMSM station N1 at 5 cm depth, the station indicates dry and wet conditions for 40 and 80 cm respectively but close to RMSM conditions for 10 and 20 cm depth. The RMSM station N2 at 10 cm depth shows a relatively dry condition for 5 cm depth but observations for other depths are not available. RMSM station C2 at 20 cm indicates MSM close to RMSM for depth 5 cm, 10 cm and 40 cm. As such, identification of a RMSM station for certain probe depth not necessarily implies that such station indicates the RMSM over the area.

Inter-comparison of MRD plots from 5 cm depth to 80 cm depth shows that the range of box values becomes smaller and closer to the box value of the RMSM station (Table 2). The MRD plot at 5 cm depth shows that lowest and highest MRD values differ by −51% (N9) and +36% (N11) with reference to the RMSM, whereas the MRD at 20 cm depth shows that MRD values differ by −13% (N10) to +20% (N5). Also, higher temporal variability is observed at shallower depth in comparison to deeper depth. At 80 cm depth, the range of MRD values is very small (close to zero) and indicates that variability is irrespective of the location in the area. Moreover, the small whiskers observed at this depth indicate low temporal variability. For further analysis, we considered the field measured values for comparison in an absolute sense. We found yearly averaged soil moisture of 0.30, 0.31, 0.33, 0.22 and 0.20 m^3^·m^−3^ for the RMSM station at 5, 10, 20, 40 and 80 cm depth, respectively. It indicates that on annual basis, soils at depths of 40 and 80 cm are dryer than soils at shallower depth. It suggests that much infiltration water is stored at shallow depths (5–20 cm), thus causing the relative large differences in MRD across the network stations at 5 cm and 10 cm in particular.

**Figure 3.**
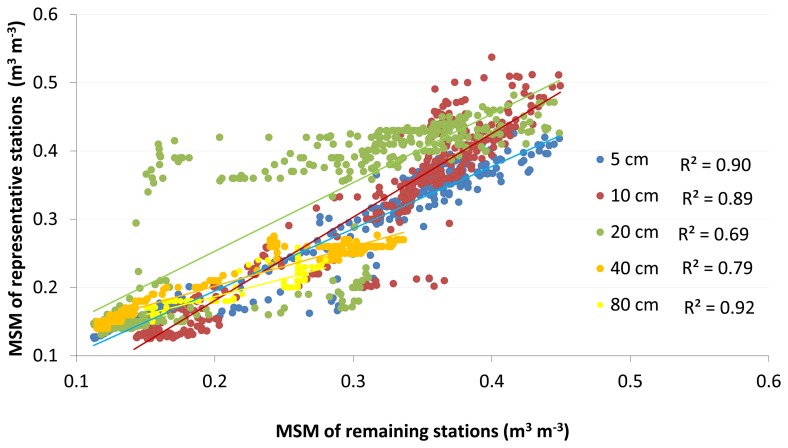
Coefficients of determination (R^2^) between daily averaged observations at the respective RMSM stations and daily averaged MSM of the remaining network stations.

To evaluate the relationship between daily average soil moisture of the RMSM station and the corresponding daily MSM of the remaining stations of the network, a scatter plot (Figure 3) is constructed. Plots are shown for all five probe depths and serve to evaluate how well the network average observations relate to observations of the RMSM station. Coefficient of determination (R^2^) ranges between 0.69–0.92. R^2^ values higher than 0.75 are indicated at 5, 10, 40 and 80 cm depth. We note that R^2^ values compare well to values reported in [24–26] and suggest that observations by the RMSM stations quite well indicate the means of the sample that is assumed to represent area MSM. The lowest R^2^ value (0.69) is found at 20 cm depth, which is caused by overestimation of MSM by the selected RMSM station at this depth (see Figure 3). At 5 cm depth, most scatter points are close to the fitted linear regression line, with largest underestimations in the 0.2–0.3 MSM value range. At 10 cm depth, the RMSM station overestimates the area MSM at higher values (0.37–0.45) and underestimates it in the 0.30–0.37 MSM value range. MSM in the area decreases at 40 and 80 cm depth with highest value of 0.33 and 0.27 respectively. High R^2^ values suggest that variability of area MSM at respective probe depths can be represented by the selected RMSM station.

### 4.2. Correlation Analysis

**Figure 4.**
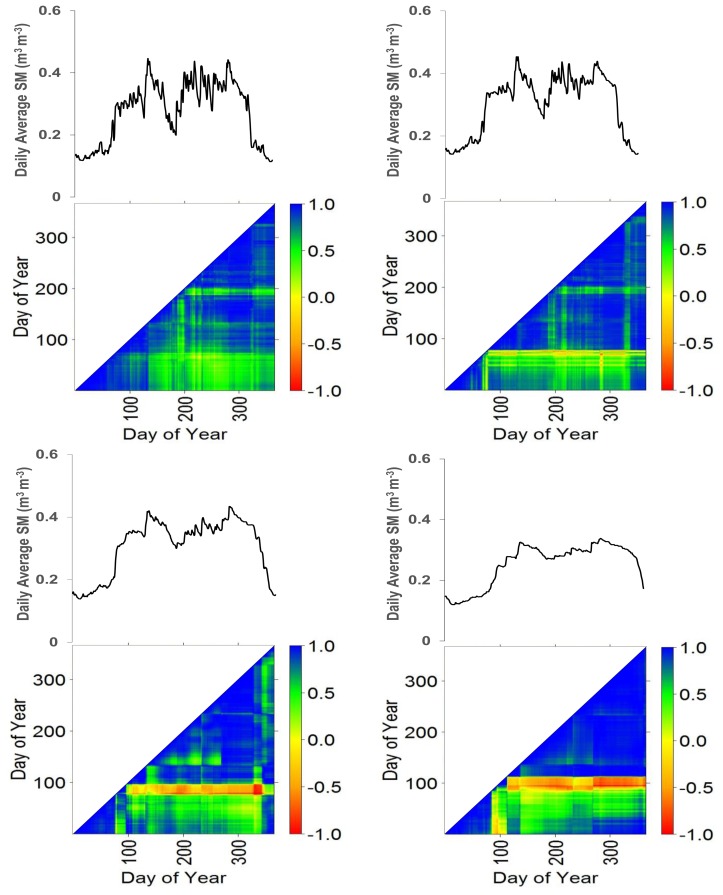
Triangular matrix of Pearson correlation coefficient (Equation (5)) at respective depth by day of year (day 1 is January 1st, 2009).

Figure 4 shows results of Pearson correlation analysis (Equation (5)) that serves to indicate temporal persistence of measurements. For each depth, the distribution of daily average moisture content over time is plotted to evaluate how moisture storage affects temporal persistence of the measurements in the time domain. When inter-comparing contents for correlation plots at 5 cm to 40 cm, results indicate that variability of moisture reduces with depth. We note that this is also indicated by the MRD plots in Figure 2. The more gradual changes over depth particularly apply to time instants at which large increases and decreases of moisture are observed at 5 cm depth (e.g., at days 80, 130, 200 and 300). Further, a comparison of graphs of the daily average moisture for the respective probe depths shows time delays in increase of moisture over depth that presumably results from infiltration, root zone flow processes and water storage. Correlation values in general increase when moving from 5 cm to 40 cm both for small time windows (<10 days) but also for periods covering months.

**Figure 5.**
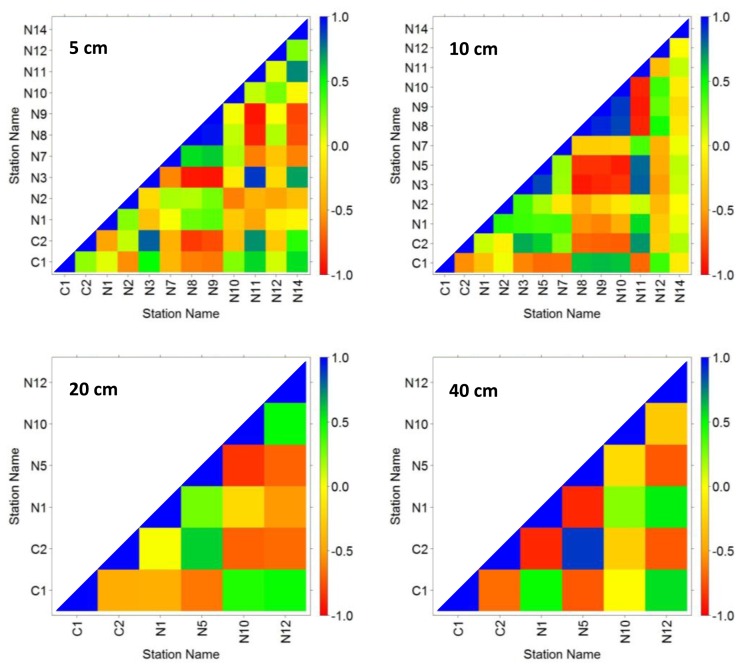
Triangular matrix of Pearson correlation coefficient [Equation (6)] between stations at respective probe depth (day 1 is January 1st, 2009).

Pearson correlation plot in Figure 5 (Equation (6)) serves to indicate correlation of measurements across the network stations. At 5 and 10 cm depth, observations from N8 and N9 stations are well correlated to other stations (for instance, correlation value near −1 are observed for stations N8 and N9 with stations C2, N3, N11 and N14). Similarly, a relatively high correlation is indicated for stations N10 and N5 with stations at 20 and 40 cm depth respectively (for instance correlation near −1 was observed for station combinations N10-N5, N10-C2, N5-C1, N5-N1, N5-N12). Further, correlation of few stations (e.g., N8, N9, N10 and N5 at 5, 10, 20 and 40 cm depth respectively) with other network stations is higher than with the RMSM station at each respective probe depth. The low correlation values at most of the stations suggest low persistent moisture patterns. This indicates relatively large changes over time and suggests that moisture patterns cannot be considered temporally stable. Correlation values between stations in general revealed low persistence of average daily soil moisture, which presumably is caused by the large inter-station distances of the network with large scale topographic variability with valleys and hillslopes.

Pearson's correlation plots (Figure 5) are compared to assess persistence of moisture patterns across the stations for respective depths. Since the number of probes at respective depths was unequal, such would introduce bias to the outcome of the analysis. To avoid bias, plots that had equal number of probes are compared which applied to 5 cm and 10 cm depth and to 20 cm and 40 cm depth. Results showed that correlation coefficients increase over the respective depth zones and indicates that the spatially persistent moisture patterns increase as we move from 5 cm to 10 cm and from 20 cm to 40 cm.

**Figure 6.**
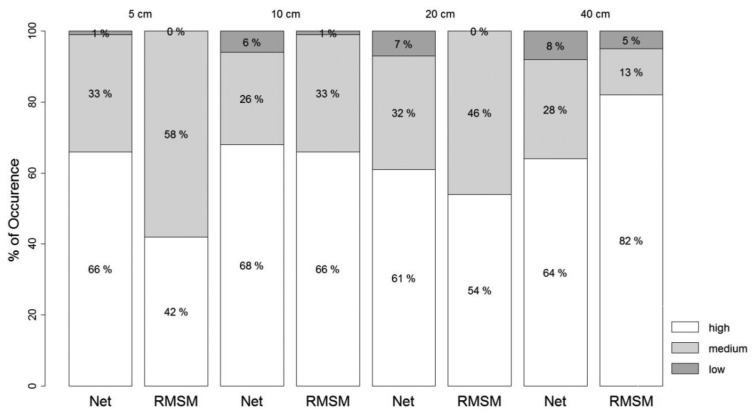
Bar plot showing occurrence (%) for high, medium and low correlation at 5 cm, 10 cm, 20 cm and 40 cm depths. For high correlation |*r_j_*,*_j_*_′_| > 0.7, for low correlation |*r_j_*,*_j_*_′_| < 0.3.

To further evaluate temporal persistence, we assessed occurrence in % of the high, medium or low correlation classes (Figure 6). Occurrence is shown for the entire network and the RMSM station at each respective probe depth to assess how persistence of measurements over time is represented by the RMSM station with reference to the network.

Bar graphs show relatively high occurrence of the high correlation class for both the network and the RMSM station at all probe depths. The average occurrence for high correlation over all depths was found to be 61% and 64% for the RMSM station and network average stations respectively. It suggests that the patterns of both the network average and the RMSM station are persistent over time. To further assess how well temporal patterns are represented by the RMSM station, we inter-compared occurrence at respective depths. Results at 5 cm show largest deviation between the network and the RMSM station with lowest occurrence of high correlation observed among all depths. At 10 cm depth, high % of occurrence (68% and 66%) of the high correlation class was found for both the network and the RMSM station respectively. It indicates that the RMSM station and network equally well represents temporal persistence. A higher value for both occurrences is only observed at 40 cm for the RMSM station, which by itself is not surprising given the outcomes of the temporal stability concept. Overall, it suggests that the selected RMSM station at 10 cm depth best represents the temporal persistence as compared to the RMSM stations at other probe depths.

**Figure 7.**
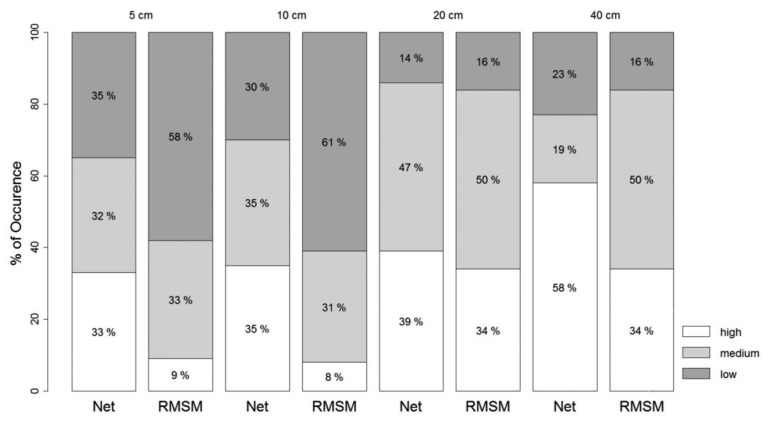
Bar plot showing occurrence (%) for high, medium and low correlation at 5 cm, 10 cm, 20 cm and 40 cm depths. For high correlation |*r_i_*,*_i_*_′_| > 0.7, for low correlation |*r_i_*,*_i_*_′_| < 0.3.

Figure 7 shows a bar plot for occurrence in % of high, medium and low correlation classes when persistence is evaluated across the network stations. The occurrences are calculated at each probe depth for all network stations and the RMSM station. Results show that for all four depths, the occurrence of the high correlation class for the RMSM station is lower than the network. At shallow depth (5 and 10 cm), the percentage of high occurrence for RMSM stations are less than 10% and much smaller than the network values (33% and 35% respectively). It indicates that the RMSM station poorly represents persistence of moisture patterns by the network. Results for 5 cm and 10 cm compare well but are quite different from results at 20 cm and 40 cm. At these depths much higher occurrence is observed for both the network and the RMSM stations, which when inter-compared, only is marginally smaller for 20 cm. Moisture variability at increasing depth reduces with more gradual changes of moisture that contributes to the high % occurrence of the high correlation class. We note that occurrence values of the high correlation class in Figure 7 are much lower than Figure 6. It indicates that correlation in the time domain is much higher than in the space domain as possibly caused by the large inter-station distances.

### 4.3. RMSM Pixel

**Figure 8.**
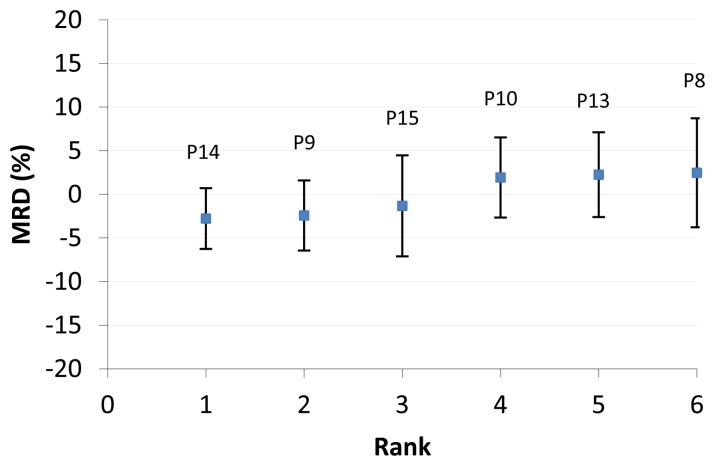
MRD plot for the screened AMSR-E VUA pixels (descending overpass) Pixel numbers are shown in Figure 1.

After selecting the RMSM station, the temporal stability analysis (Equations (1–4)) was applied to the satellite images of the descending overpasses of the AMSR-E satellite. We constructed a MRD plot (Figure 8) for the screened AMSR-E VUA pixels (Table 1) that serves to evaluate if a RMSM pixel can be identified.

The MRD plot shows that pixel P15 has MRD value −1.3% that is closest to zero with SD(MRD) of 5.8%. Following the principles of the temporal stability concept, we identify this pixel as the RMSM pixel that should indicate the satellite-based RMSM. The MRD plot in Figure 8 shows MRD and SD(MRD) values for all pixels. Negative MRD values indicate dryer pixels than the mean value whereas positive MRD values indicate the wetter pixels. Also the whiskers for the pixels show variation (although relatively small) and suggest that certain pixels may be characterized by relatively large moisture variability, whereas other pixels have much lower variability. With respect to objective two of this study, results indicate that the temporal stability concept can effectively be applied to a series of satellite images and that a RMSM pixel (here P15) can be identified. To analyze if satellite validation may benefit from applications, we inter-compared MRD plots of Figure 2 and Figure 8 (station data and satellite data, respectively). Somehow surprising was that the RMSM pixel did not overlay the location of the RMSM station at shallow depth (5 cm or 10 cm) so to evaluate how well the RMSM pixel indicates RMSM as observed by the network.

MRD plots (Figure 2 and Figure 8) indicate that temporal stability in general is higher for stations with relatively dry field conditions. Driest field conditions for respective probe depths are shown for stations N9 at 5 and 10 cm depth; N10 at 20 cm depth and N1 and N12 at 40 cm and 80 cm depth respectively. Figure 8 shows that P14 and P8 are the driest and wettest pixels respectively, although the difference in MRD only is relatively small. To assess how well temporal variability is represented by the satellite observations, the SD(MRD) values for the driest and wettest stations and pixels are inter-compared (Table 3). Results showed that values at shallow probe depth for both wet and dry conditions are much larger than pixel based counterparts. SD(MRD) for dry stations reduces from 14% at 5 cm to 9% at 40 cm and from 16% (5 cm) to 8% (40 cm) for wet stations. SD(MRD) values for pixels only are 3% and 6% for dry and wet conditions, respectively, and are lower than the lowest SD(MRD) station values. The inter-comparison indicates that temporal stability by network stations only is poorly represented by the satellite images and infers that satellite images indicate much too high temporal stability.

### 4.4. Time Series Analysis

**Figure 9.**
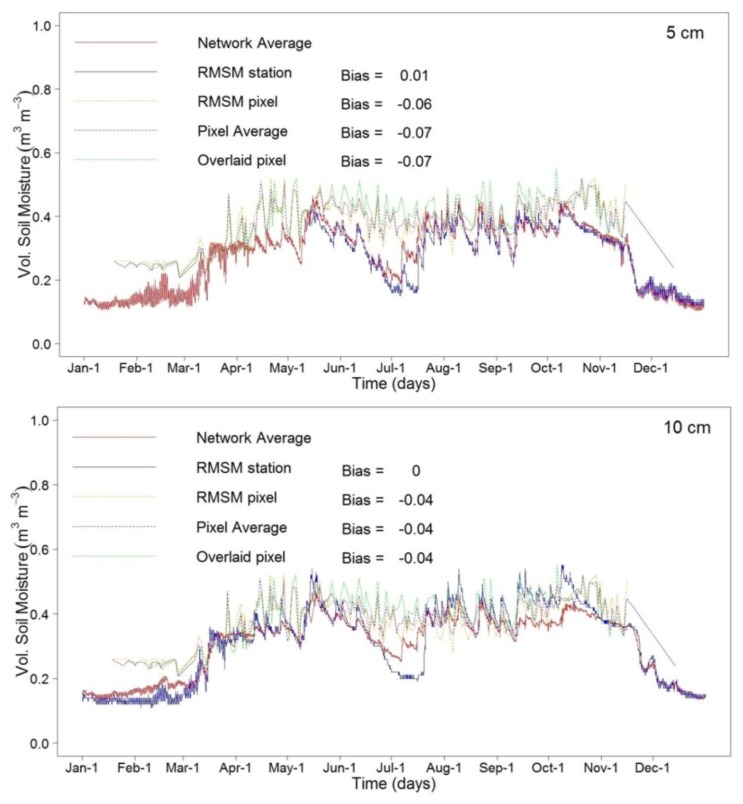

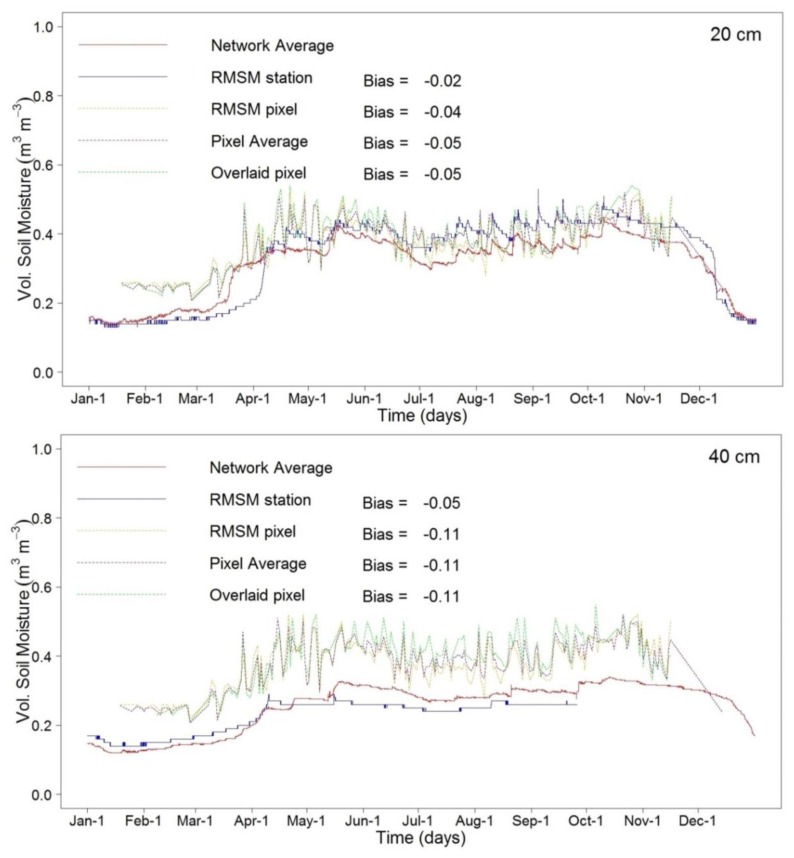
Time series comparison of soil moisture observation from network average (*i.e.*, the bench mark) to the RMSM station, RMSM pixel, pixel average and the satellite pixel that overlays the RMSM station.

To further evaluate how well network observations are represented by satellite images, we compared daily time series from the network to four series that are from the RMSM station, the RMSM pixel, the average of the image pixels and the pixel that overlays the RMSM station (*i.e.*, the overlay pixel). For comparison, we consider the network average at the respective probe depth as the benchmark. The daily average was computed by taking the arithmetic mean of *in-situ* moisture observations collected for each day. For comparing observations at the satellite overpass time, we used the *in-situ* observations closest to the satellite overpass time to rule out that rain events disturb the relation. We note that for all four depths time series of the RMSM pixel and the average of the image pixels are the same. Visual inspection of Figure 9 shows similar patterns for all the satellite-based estimates although the satellite-based time series overestimate the probe-based time series. Small deviations between the RMSM pixel and the image pixel averages suggest that the average of the image pixels is well presented by the RMSM pixel.

Visual inspection of the moisture time series of the network average and the RMSM station at 5 cm shows a good match in general except in July where the network indicates higher values. Both time series, however, show lower moisture compared to time series of the satellite-based RMSM pixel and the average of the image pixels. Further, satellite-based time series show much lower temporal variability compared to the network average and the RMSM station observations. Time series of the overlay pixel show values that largely deviate from the RMSM station, the RMSM pixel and the average of the image pixels. Further, the overlay pixel shows higher moisture values and indicates higher variability. We note that both RMSE and Bias have similar value for the RMSM pixel, the average of the image pixels and the overlay pixel.

Visual inspection of the time series at 10 cm depth shows several periods where the RMSM station observation deviates from the network observation. Similar to the comparison at 5 cm, the RMSM station underestimates the network average for the month of July, but for the period August–November, overestimation is indicated. For this period, however, a fair match is shown for the RMSM station and all three satellite-based time series. At deeper depth (20 cm and 40 cm) this match deteriorates. Also the network averages show increasing deviation from the RMSM station for essentially two periods. For the period January–April, the RMSM station shows underestimation whereas the RMSM station overestimates the network average for the remaining period. Variability for both time series is relatively low at 20 cm depth and, actually, is much lower than variability indicated by the satellite-based time series. We note that similar patterns are indicated at 40 cm probe depth. Patterns, however, are more pronounced with much lower temporal variability for the probe-based time series. Both the RMSM station and network average time series underestimate the satellite-based time series for all days of the year. Therefore observations at 20 cm and 40 cm are not suitable for satellite validation purposes. Most suitable for satellite validation are the observations at 10 cm depth by the fair matches between the RMSM station, the network average and the satellite-based time series for the period August–November. A complicating factor to all comparisons is the period June–July where a pronounced decrease of moisture storage is indicated by the probe-based time series. This decrease only is poorly represented by the satellite with largest deviations at shallow probe depths (5 cm and 10 cm).

**Figure 10.**
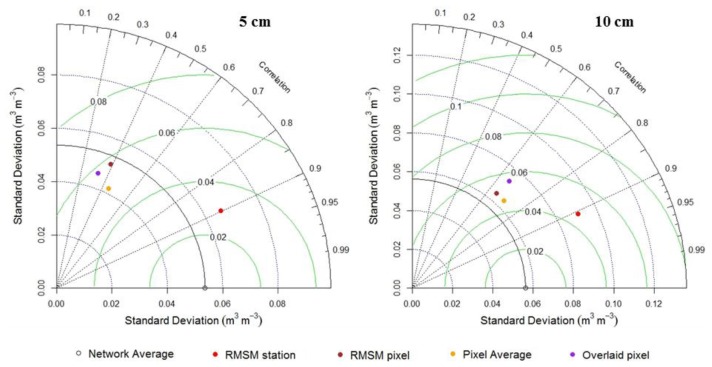
Taylor diagram illustrating statistics of the comparison between RMSM station, RMSM pixel, pixel average, pixel overlay on the RMSM station and the network average (the bench mark). The azimuthal angle represents correlation coefficient; radial distance represents standard deviation (m^3^·m^−3^) of the soil moisture time series and green contours represent RMSE (m^3^·m^−3^).

Further results on time series analysis are shown by Taylor's diagram [36], that in this study, aims to characterize the statistical relationship between the time series of the RMSM station, RMSM pixel, pixel average and overlay pixel, (*i.e.*, test sample) and the network average (*i.e.*, bench mark). Figure 10 summarizes how closely test samples match to the bench mark. The similarity between patterns is assessed in terms of RMSE, correlation and the standard deviation (amplitude of the variations).

The relative merits in terms of statistics for each test sample can be inferred from Figure 10. The green contours represent RMSE values between the sample patterns and the bench mark patterns and are proportional to the point that represents the network average on the x-axis. The standard deviations of the test and bench mark patterns is shown by dotted and continuous black lines, respectively, and are proportional to the radial distance from the origin. The azimuthal angle represents the correlation coefficient value. Test sample patterns which agree well with the bench mark pattern lie close to the point marked on the x-axis. These patterns indicate relatively high correlation and low RMSE [36].

We plotted the Taylor diagram for 5 and 10 cm depth following results from previous analysis. At both depths, correlation around 0.9 is found for the RMSM stations. For other time series, correlation varies from 0.33–0.45 and from 0.63–0.73 for 5 and 10 cm, respectively. At 5 cm, lowest RMSE (0.03 m^3^·m^−3^) is found for RMSM station that in case of other patterns varies from 0.05–0.06 m^3^·m^−3^. RMSE varies from 0.04–0.06 m^3^·m^−3^ at 10 cm with lowest values for RMSM station and pixel average observations. The standard deviation of RMSM station at 5 and 10 cm (about 0.07 m^3^·m^−3^ and 0.09 m^3^·m^−3^ respectively) is larger than the network average observations at these depths which is indicated by the continuous arc near 0.06 m^3^·m^−3^. For other time series observations, lower and higher standard deviation than the network average is observed at 5 and 10 cm, respectively. Higher standard deviation of RMSM stations at 5 and 10 cm shows larger variation of amplitude than the network average observation.

In [37–39] it is shown that accuracy of satellite data relies in the reproduction of the soil moisture temporal variability and therefore its accuracy should not be checked on absolute values. Therefore, the accuracy of satellite data is evaluated with the correlation coefficient instead of RMSE and/or SD. Figure 10 shows that all test samples have highest correlation values at 10 cm depth. It indicates that observations at 10 cm are most suited for satellite-based soil moisture validation in the Maqu area.

Results of our comprehensive analyses are based on widely accepted and independent methods that all indicate that field observations at 10 cm depth best match to the satellite observations. This outcome was somewhat surprising since penetration depth of the microwave signal commonly is considered to be less than 5 cm. We refer to [4] on the issue of the penetration depth where it is stated that a measurement depth is not a constant, but it is related to the moisture content and to the operational frequency of the sensor. In [4], surface roughness and vegetation cover are indicated as important factors that affected penetration depth of the MW signal. Our study area is flat barren land where surface roughness effects and attenuation from vegetation are not pre-dominant. This does not rule out deeper penetration and may add to our findings for the Maqu area.

Critical to applications of the temporal stability concept is that the sample size of observations should be sufficiently large to represent the natural variability of soil moisture. The minimum sampling time is identified by evaluating the progression of MRD and SD(MRD) values in the time dimension. In our study MRD and SD(MRD) values did not change markedly after 11 months and is only just within the length of the available time series that covered 12 month. To better substantiate on findings of the temporal stability concept we advocate usage of a much longer period.

## 5. Conclusions

Results of temporal stability analysis in this study showed that for each probe depth, a specific RMSM station can be identified. However, the identified RMSM station differs at each depth. Analysis indicated that for all depths, catchment MSM is well represented by the RMSM station. Based on Pearson's correlation analysis, we found that correlation in the time domain is much higher than in the space domain but appears plausible by the large inter-station distances. Application of the temporal stability concept, pearson's correlation analysis and results shown by Taylor's Diagram indicate that observations at 10 cm probe are most suited to be used for soil moisture satellite validation in the Maqu area. Results of application of the temporal stability concept to a time series of satellite images showed that a pixel indicting RMSM can be identified. This RMSM pixel, however, didn't overlay a RMSM station at any of the probe depths so we could not show that the RMSM pixel overlays a RMSM station. Results indicate that the AMSR-E VUA images have relatively low temporal variability as indicated by small values of MRD and SD(MRD). Values for the satellite pixels are relatively small and actually much smaller than probe-based MRD and SD(MRD) values at shallow depth (5 and 10 cm). Deviations are not systematic over the observation period so conclusions on bias effects are not drawn. A comparison between network averaged and image averaged time series shows that the satellite images in particular have difficulty to represent moisture conditions under dry conditions. Time series analysis showed that network average moisture contents best match with observation time series at 10 cm probe depth. This is also indicated in findings on the RMSM station where we showed that the 10 cm observation depth is best suited to represent temporal persistence (see Figure 6) and thus MSM. Our finding that 10 cm depth is best suited is somewhat unexpected since, *a priori*, we anticipated that 5 cm would be more suited. Possible reasons could be the specific topographic, land cover and pedology of the Maqu area that is flat barren and would allow penetration depth beyond 5 cm. With respect to the relative short time series used for this study, we advocate to use time series much longer than 12 months to better substantiate on findings of the temporal stability concept to the Maqu area.

Based on the extensive but complementary analysis in this study, we conclude that probe observations at 10 cm depth are best suited to serve validation of the AMSR-E VUA images in Maqu area. We note that we compared observations directly so we ignored aspect of profile or root zone moisture. Further, probe network densities are unequal for respective depths so inter-comparison of correlation results rely on different samples which may have affected our findings. In this study, we report on a first application of the temporal stability concept to a series of satellite images. Results in this study show that the concept is very well applicable in satellite-based moisture assessments. We note that applicability to a time series of images should be tested more widely, particularly by considering profile soil moisture. Overall, we conclude that validation of satellite moisture products may benefit from application of the temporal stability concept. Applications should preferably be tested for more dense networks and larger number of satellite pixels.

## Figures and Tables

**Table 1.** *In-situ* stations that are overlain by a pixel.

**Stations**	**Pixel**
C1, N1, N2, N3, N14	P8
N7	P9
N8, N9, N10	P10
C2, N5	P13
N11, N12	P14
No stations available	P15

**Table 2.** Minimum (Min) and Maximum (Max) values of MRD and SD(MRD) at 5, 10, 20, 40, 80 cm depth at the Maqu network stations.

**Depth (cm)**	**MRD (%)**			**SD(MRD) (%)**		

	**Min**	**Max**	**Range**	**Min**	**Max**	**Range**
5	−51	+36	87	8	36	26
10	−34	+34	68	5	18	13
20	−13	+20	33	5	15	10
40	−19	+23	42	8	13	5
80	−12	+14	26	5	10	5

MRD is Mean Relative Difference; SD(MRD) is standard deviation of the MRD.

**Table 3.** SD(MRD) of the driest and the wettest stations/pixels at 5, 10, 20 and 40 cm depth.

**Depth (cm)**	**Driest Station/Pixel**		**Wettest Station/Pixel**	

	**Name**	**SD(MRD)%**	**Name**	**SD(MRD)%**
5	N9	14	N11	16
10	N9	13	N5	15
20	N10	8	N5	9
40	N1	9	N5	8
-	P14	3	P8	6
